# Ontological deskilling: clinical expertise and the changing disease object in precision medicine

**DOI:** 10.1007/s11019-026-10377-9

**Published:** 2026-07-08

**Authors:** Anastasios E. Germenis

**Affiliations:** Department of Immunology and Histocompatibility, School of Medicine, University of Thessaly, Larissa, Greece

**Keywords:** Clinical expertise, Precision medicine, Philosophy of medicine, Disease classification, Artificial intelligence, Deskilling, Uncertainty

## Abstract

Concerns about physician deskilling in AI-enabled medicine usually ask how automated systems substitute for, attenuate, or distort clinical capacities. This article argues that precision medicine introduces a distinct object-side problem. Its dominant classificatory operation is not simply finer nosological classification but stratification for action: patients are grouped and regrouped for treatment selection, risk allocation, surveillance, trial eligibility, and prediction. In doing so, precision medicine fragments established disease categories, connects conditions across former boundaries, temporalizes disease into trajectories and thresholds, and produces actionable risk states and algorithmic subgroups. If clinical expertise is acquired through relatively stable nosological categories, then preserved skills may become insufficient when those categories no longer organize the clinical object in the same way. I call this failure of fit ontological deskilling. The article distinguishes it from technological deskilling, diagnostic uncertainty, overdiagnosis, medicalization, plastic diagnostics, and ordinary adaptive-learning demands. Drawing on Canguilhem, Mol, phenomenological intentionality, and accounts of expertise, it argues that ontological deskilling changes the conditions of clinical judgment, uncertainty, and responsibility. The appropriate response is critical nosological awareness: the ability to use disease categories competently while recognizing their contingency, plurality of functions, and susceptibility to transformation.

## Introduction

When the National Research Council outlined its vision for precision medicine in 2011, it did not present the enterprise merely as a way of tailoring treatment within existing diagnostic categories. It described a program for constructing a Knowledge Network of Disease and, on that basis, “a New Taxonomy of Disease” grounded in molecular biology and integrated data infrastructures (National Research Council 2011). Precision medicine was therefore announced from the beginning as a nosological project. Its ambition was to reorganize the categories through which disease is identified, ordered, and made actionable.

Recent work in the social studies and philosophy of disease classification has shown that this ambition is already reshaping medicine. Wadmann (2023) situates precision medicine within the longer historical and social organization of disease classifications, while Green et al. (2022) describe the emergence of “plastic diagnostics,” in which disease categories become more fine-grained, revisable, and responsive to evidential, technological, and regulatory pressures. The present article builds on this literature but shifts the analytic focus. Its concern is not primarily how classifications change, but what such change does to the structure of clinical expertise. If physicians acquire their perceptual, inferential, and practical capacities within historically stabilized disease categories, then the plasticity of those categories has consequences not only for nosology but for the adequacy of clinical competence itself.

For clarity, the “disease object” in this article does not mean a metaphysically simple entity lying behind clinical practice. It refers to the clinically operative object toward which recognition, explanation, prognosis, communication, and intervention are directed. A disease object may be a classical diagnosis, a molecular subtype, a residual molecular signal, a risk state, an algorithmic subgroup, or a coordinated assemblage of these. The term is deliberately pragmatic: it names the object around which clinical work is organized, not an ultimate substrate. Following practice-oriented accounts of medical ontology, the relevant question is how such objects are constituted, stabilized, coordinated, and made actionable in clinical work (Mol 2002).

Nosology is therefore not merely a system of names. It is one of the cognitive infrastructures through which physicians learn to see and to act. In medical education, disease categories are gradually transformed from explicit propositions into illness scripts, perceptual habits, and patterns of rapid recognition that organize clinical attention before deliberate analysis begins (Schmidt and Rikers 2007). The category is not simply something the physician knows; it helps shape the form within which she knows. This is why a deep transformation of nosology can become a transformation of clinical cognition itself.

The point is not that clinicians consciously consult a taxonomy before every judgment. Quite the contrary: once expertise has been acquired, categories often withdraw from explicit attention. They structure the field of salience, determine which findings count as relevant, organize differential diagnosis, and connect recognition to prognosis and action. This tacit dimension is precisely what makes nosological transformation clinically important. A change in classification is not simply an external revision of labels; it can alter the background conditions under which clinical perception and reasoning operate.

The contemporary discussion of physician deskilling has understandably focused on artificial intelligence. As AI systems enter imaging, pathology, documentation, triage, prediction, and decision support, concerns have arisen that they may erode the competencies they are designed to support. Existing accounts explain deskilling through substitution, diminished practice, cognitive offloading, automation bias, or the reduction of the physician to a validator of algorithmic outputs (Goh et al. 2024; Heudel et al. 2026; Natali et al. 2025; Oettl et al. 2026). These analyses identify a real vulnerability. Yet they typically ask how new systems alter the physician’s capacities, not whether the objects of those capacities are themselves changing.

This article argues that precision medicine produces a second vulnerability: one that originates on the side of the object rather than only on the side of the agent or tool. The same scientific and computational developments that have generated new tools for decision-making have also destabilized the nosological order within which clinical expertise was formed. Established diseases are molecularly fragmented; conditions once considered separate are connected through shared pathways or actionable alterations; risk states acquire quasi-nosological force; and algorithmic systems generate clinically useful groupings that may lack the semantic structure of classical diagnoses. The result is an increasingly heterogeneous landscape in which disease objects differ in epistemic provenance, temporal orientation, clinical authority, and relation to the patient’s lived experience.

The article introduces the term ontological deskilling for the erosion or insufficiency of clinical competence that arises when the disease object itself has been reconstituted. This is not ordinary ignorance, not mere diagnostic uncertainty, and not simply the failure to keep up with new information. It is a failure of fit between a competence acquired in relation to one object-structure and a clinical field in which that object-structure has changed. The physician may continue to possess the relevant skill by conventional standards and yet find that the skill no longer answers to the form in which disease now presents itself. Because this failure of fit affects what clinicians can responsibly know, communicate, and decide, the argument carries normative implications that go beyond a diagnosis of clinical difficulty. It bears on what is owed to patients and on what institutions, not only individual clinicians, must do in response.

The term “deskilling” is retained deliberately. The problem is not simply that clinicians must learn new facts or add molecular and computational information to an otherwise stable diagnostic repertoire, nor is it that inherited skills become obsolete or are cleanly replaced by new ones. Rather, a competence that once counted as adequate may become insufficient because the object to which it was attuned has been displaced. This is not a general complaint about innovation. Medicine has always revised its categories. The more specific claim is that precision medicine makes the instability of disease objects recurrent, operational, and clinically consequential in domains where expertise was historically acquired through relatively stable categories.

Two clarifications are needed at this point. First, the AI applications most directly implicated in the object-side transformation analyzed here are those embedded in the epistemic infrastructure of precision medicine itself, namely clinical decision support systems, predictive and prognostic models, machine-learning-based stratification, and computational subtype discovery. These do not only mediate the physician’s access to a pre-existing disease landscape; they participate in the constitution of new clinical objects, including algorithmic subgroups, risk states, predicted trajectories, and actionable molecular signals that may or may not align with classical nosological categories. Generative and conversational AI, as well as AI-driven documentation, raise their own important concerns about communication, professional formation, and clinical reasoning, but they figure only peripherally in the present argument because their relation to the reconstitution of the disease object is more indirect. Second, AI-enabled medicine and precision medicine are neither synonymous nor independent. Precision medicine names a broader epistemic and clinical regime that reorganizes disease around molecular variation, stratification for action, and integrated data infrastructures. AI, in the relevant sense, is one of the principal computational means through which this regime operates. The ontological deskilling thesis is therefore not a thesis about AI in general, nor a thesis about precision medicine in general, but about a specific intersection, namely the form of clinical insufficiency that emerges when expertise trained on classical nosology must operate within a domain in which the disease object is being reconstituted by precision medicine, often through AI-mediated processes.

The argument proceeds in five steps. The first section reviews existing accounts of physician deskilling and identifies their usual focus on the doctor-machine relation. The second analyzes precision medicine as a transformation of the disease object through molecular fragmentation, actionable risk, temporalization, and computational representation. The third defines ontological deskilling and distinguishes it from adjacent concepts. The fourth examines its implications for clinical judgment, uncertainty, and responsibility, and develops the normative case for why the situation calls for collective and institutional response, not only individual adaptation. The final section argues that the appropriate response is critical nosological awareness: a form of adaptive clinical expertise that preserves the practical value of disease categories while rendering their contingency, multiplicity, and revisability visible.

## Existing accounts of physician deskilling

Artificial intelligence has entered medicine as a technology of considerable promise. Across imaging, pathology, clinical documentation, prediction, triage, and decision support, AI applications are presented as ways to improve efficiency, standardization, and, in selected settings, diagnostic accuracy. They are often described as augmentative rather than substitutive tools: by automating repetitive or administratively burdensome tasks, they may release physicians’ time for more complex reasoning and patient-facing care (El Tarhouny and Farghaly 2026; Khan 2023; Oettl et al. 2026). The literature on physician deskilling does not generally arise from a rejection of these benefits. It arises from concern that the same tools may erode capacities on which clinical work depends.

One family of accounts understands deskilling as technological substitution followed by atrophy. Skills weaken when tasks once performed directly by clinicians are delegated to automated systems. The problem is not only that a machine performs part of the work, but that trainees and practitioners lose opportunities for repetition, correction, and consolidation. Perceptual, interpretive, or procedural capacities become less robust because they are less often independently exercised. Heudel et al. describe this in terms of decreased practice, cognitive offloading, and a shift from task execution to supervisory monitoring; Natali et al. similarly present AI-induced deskilling as a broad erosion of professional expertise under conditions of automation, even where new oversight skills may emerge in parallel (Heudel et al. 2026; Natali et al. 2025).

A second family of accounts emphasizes distortion rather than simple disuse. AI may improve performance under some circumstances while fostering automation bias, over-trust, or reduced critical scrutiny of machine outputs. Clinicians may accept erroneous prompts, overvalue recommendations, or revise correct initial judgments in response to misleading algorithmic suggestions. In this sense, deskilling is not only the fading of a capacity through inactivity; it is the deformation of clinical judgment under technologically mediated dependence (Heudel et al. 2026).

A third account concerns cognitive autonomy. The worry here is that the physician’s role may gradually shift from independent clinical judgment to the passive validation of algorithmic outputs. The randomized trial by Goh et al. is instructive in this respect: physicians with access to a large language model did not significantly outperform physicians using conventional resources alone, while the model alone outperformed both physician groups (Goh et al. 2024). The finding does not show that physicians are unnecessary. It shows that the availability of a powerful tool does not automatically improve human reasoning and may expose a fragile interface between machine output and clinical judgment.

More recent discussions have broadened the concept of deskilling beyond technical competence. Heudel et al. distinguish technical, cognitive, moral, social, and semiotic dimensions; El Tarhouny and Farghaly connect deskilling to the weakening of adaptive expertise; and Oettl et al. warn against “never-skilling,” where trainees exposed too early to AI-assisted workflows may fail to acquire foundational competence at all (El Tarhouny and Farghaly 2026; Heudel et al. 2026; Oettl et al. 2026). These accounts are important because they show that deskilling is not simply manual decline. It may also involve judgment, communication, professional identity, and the capacity to reason under uncertainty.

Even in these broader versions, deskilling is usually conceptualized through the effects of intelligent systems on physicians’ capacities. The central analytic scene remains one in which a physician confronts a machine whose outputs, affordances, and efficiencies alter performance. This framing is not wrong, but it is incomplete. It leaves largely unasked whether the clinical object toward which performance is directed remains stable.

This limitation should not be overstated. Some deskilling accounts already recognize that AI reorganizes social roles, moral responsibility, and semiotic sensitivity. The criticism is narrower: these accounts rarely make the ontology of the disease object the principal object of analysis. They tend to ask whether physicians will continue to see, infer, decide, and communicate well when machines mediate their work. They less often ask whether the “thing” to be seen, inferred, decided about, and communicated has itself been reconstituted by the same scientific and computational developments that produced the machine.

This distinction matters for evaluation as well as theory. A training program designed only to prevent technological deskilling will test whether clinicians can still perform a task without assistance. A training program designed to address ontological deskilling must ask a different question: can clinicians explain how patients previously grouped under the same diagnosis may become different clinical objects when stratified by molecular mechanism, risk trajectory, algorithmic cluster, or therapeutic target? The two assessments may overlap, but they do not measure the same vulnerability.

### Precision medicine and the changing disease object

Precision medicine has emerged at the intersection of post-genomic biomedicine, health big data, computational medicine, and clinical decision support. This intersection matters for the present argument because AI does not enter clinical practice on top of an unchanged disease landscape; it enters as part of the same computational and biological infrastructure that is reorganizing that landscape. Its clinical promise is individualized intervention: the selection, timing, or avoidance of treatment according to biologically and computationally specified differences among patients. For that reason, its dominant classificatory operation is not classification in the traditional nosological sense, but stratification for action. Precision medicine groups and regroups patients to identify therapeutic targets, predict response, allocate risk, determine trial eligibility, or guide surveillance. It is therefore a regime for producing and operationalizing disease knowledge through molecular data, interoperable infrastructures, knowledge graphs, and algorithmic systems capable of acting on heterogeneous datasets (Hulsen et al. 2019; Johnson et al. 2021; National Research Council 2011). It changes not only what physicians know about diseases, but also the purposes for which disease-related categories are formed and used.

Traditional disease classifications have served several functions at once: diagnosis, prognosis, communication, research aggregation, regulatory authorization, reimbursement, and therapeutic decision-making. In relatively stable nosological regimes, these functions can appear to converge on a single disease entity. Precision medicine weakens that convergence because its clinical aim is not primarily to produce a more refined taxonomy, but to sort patients into actionable groups. The grouping that best predicts treatment response may not be the grouping that best explains symptoms, supports reimbursement, organizes patient identity, or anchors epidemiological comparison. The resulting problem is not greater classificatory resolution alone; it is a partial dissociation between classical nosological classification and intervention-oriented stratification.

This transformation raises a familiar risk of biological reductionism. Although precision medicine often presents itself as more individualized, its current implementation remains heavily organized around biological and molecular data, especially genomics and other omics. Delpierre and Lefèvre argue that contemporary precision medicine remains largely aligned with a biomedical model unless environmental, socio-economic, psychological, and biological determinants are integrated within a broader biopsychosocial framework (Delpierre and Lefèvre 2023). This point matters for the present argument because a molecularly precise disease object is not necessarily a personally meaningful clinical object. The physician must coordinate refined biological classifications with the person for whom risk, illness, treatment, and future possibility acquire practical meaning (Abettan and Welie 2020; El-Alti et al. 2019).

Precision medicine also reactivates a central question in the philosophy of medicine: what is a disease? Naturalist accounts, most influentially associated with Boorse, treat disease as objective biological dysfunction; normativist accounts, represented by Nordenfelt, connect disease to harm, incapacity, or the frustration of vital goals; hybrid accounts such as Wakefield’s harmful dysfunction analysis combine dysfunction with harm (Boorse 1977; Nordenfelt 2007; Wakefield 1992). Precision medicine does not settle these debates. It reopens them in clinical practice by making molecular variation, risk thresholds, residual signals, and predicted trajectories into objects of medical attention.

This reopening of the disease question should also be situated in relation to Sadegh-Zadeh’s analytic philosophy of medicine. Long before current debates on precision medicine, Sadegh-Zadeh argued that health, illness, disease, and disease entities are better understood as fuzzy rather than sharply bounded concepts, and that nosology requires a language capable of accommodating degrees, borderline cases, and non-Aristotelian conceptual structures (Sadegh-Zadeh 1999, 2000). This point matters here because it already challenges the assumption that disease categories behave as crisp, classical kinds. The argument developed in this article does not adopt a fuzzy-logical reconstruction of disease concepts. Its focus is different: not the formal structure of vague disease predicates, but the way in which precision medicine transforms the objects toward which clinical expertise is directed.

The relevance of this debate for the present article is limited but important. The argument does not require choosing between naturalism and normativism. It requires recognizing that precision medicine multiplies the kinds of objects that can claim clinical significance. Some are biological abnormalities without current illness; some are risk states without present dysfunction; some are molecular mechanisms that cross established disease boundaries; some are algorithmic groupings that are actionable without being explanatory in the ordinary clinical sense. The disease debate therefore reappears not as an abstract classificatory exercise, but as a practical question about what kind of object the clinician is being asked to recognize and act upon. This redirection is itself one of the conditions of ontological deskilling. The metaphysical question about disease is no longer one the clinician can leave to philosophers, because the kinds of objects she must act upon force the question into clinical work itself.

Canguilhem’s analysis of the normal and the pathological is especially useful here. For Canguilhem, pathology cannot be reduced to statistical deviation or objective abnormality; it concerns the organism’s normativity, its capacity to establish and transform norms in relation to its milieu (Canguilhem 1991). Precision medicine shifts this problem into a new register. A pathogenic variant, residual molecular signal, or algorithmic probability may become clinically actionable before symptoms, functional impairment, or any experienced disturbance of life. The pathological may thus appear as a future-oriented, probabilistic, or technologically disclosed object before it becomes an illness in the experiential sense (Giroux 2020).

This also explains why the distinction between personalized medicine and person-centered care matters. Personalized medicine often individualizes by subdividing biological difference: variant, biomarker, pathway, risk score, or therapeutic target. Person-centered care individualizes by attending to values, narrative, social context, embodied experience, and practical meaning (El-Alti et al. 2019). Ontological deskilling becomes ethically significant when the first form of individualization generates objects that the physician can act on but cannot adequately integrate into the second. A molecularly precise object may still be clinically incomplete if it cannot be related to the life in which illness or risk must be lived.

Molecular fragmentation provides the most familiar form of this transformation. Conditions long treated as unitary disease entities are increasingly shown to contain biologically distinct processes. Breast cancer is paradigmatic: molecular profiling differentiates what had been an organ-based category into subtypes such as luminal A, luminal B, HER2-enriched, and basal-like disease, each with different biological behavior, prognosis, and therapeutic sensitivity (Perou et al. 2000; Sørlie et al. 2001). Conversely, shared molecular alterations may connect conditions that classical taxonomy placed in different domains. LMNA mutations, for example, underlie clinically diverse conditions including Emery-Dreifuss muscular dystrophy, dilated cardiomyopathy, familial partial lipodystrophy, and progeroid syndromes (Worman and Bonne 2007). Disease identity becomes dependent on the level of analysis: phenotype, mechanism, therapeutic target, prognosis, or risk.

Stratification operationalizes this shift. Rather than being treated only as instances of a disease class, patients are distributed across subgroups defined by molecular, genetic, or biomarker profiles that predict course or therapeutic response. These groupings are purpose-dependent and intervention-oriented. A stratification useful for prognosis may differ from one guiding treatment, and a biomarker relevant in one clinical context may cut across several conventional diseases in another. This is not classification at a finer grain in the traditional sense. It is classification subordinated to individualization of care: treatment selection, risk allocation, surveillance, or trial access. Tissue-agnostic therapies make the point explicit: the actionable unit of treatment may be a molecular alteration rather than an organ-based disease category (Drilon et al. 2018; Lord and Ashworth 2017). Classical categories persist as institutional and clinical references, but they no longer consistently function as the primary units of therapeutic reasoning.

The effects on the inherited classificatory landscape can be summarized in three movements. First, established disease categories are subdivided as molecular and biomarker data differentiate previously unified clinical entities. Second, they are realigned as shared mechanisms or actionable alterations connect conditions that classical taxonomy separated. Third, they become operationally plastic because groupings may be generated for different purposes: explanation, prognosis, treatment selection, trial eligibility, surveillance, or risk management. The point is not that precision medicine produces a superior classification of greater detail. It is that inherited categories become one layer within a changing field of objects, each carrying a different relation to explanation, prediction, action, and institutional use.

Precision medicine also brings into being objects that had no secure place in classical diagnostic medicine. The most important is not the molecular lesion as such, but the medically actionable state of risk. Classical diagnosis was organized around the identification of present pathology; prognosis followed from an already constituted disease. Genomic and other predictive technologies can reverse this order by classifying persons according to what they may become. Pathogenic or likely pathogenic variants detected through whole-exome or whole-genome sequencing may not identify present disease, but they can justify surveillance, cascade testing, preventive intervention, or reproductive decision-making (ACMG Board of Directors 2015; Green et al. 2013). Risk thereby acquires a quasi-nosological status.

This should not be confused with the claim that precision medicine only extends medicalization or increases overdiagnosis. Medicalization concerns the framing of phenomena as medical problems; overdiagnosis concerns the detection of abnormalities that would not have caused symptoms or harm in the absence of testing (Hofmann 2016; van Dijk et al. 2016; Walker and Rogers 2017). These concerns remain important. Ontological deskilling asks a different question: what happens to clinical expertise when the objects of classification are no longer straightforwardly present diseases, but molecular, probabilistic, temporal, or anticipatory states. The issue is not only the expansion of medicine’s jurisdiction, but the alteration of the object to which clinical competence is directed.

Minimal residual disease (MRD) makes this transformation clinically tangible and exposes most clearly the kind of object whose emergence the present argument concerns. After apparently successful treatment of a hematological malignancy, conventional criteria may indicate remission, while highly sensitive molecular assays detect residual malignant material below the threshold of clinical visibility. MRD is neither manifest disease nor simple absence of disease. It is a technologically disclosed state whose significance lies in its relation to possible relapse (Chin-Yee 2024; Heuser et al. 2021). Such objects may be therapeutically important, but they also unsettle inherited categories of recognition: the physician must judge a state situated between presence and absence, current disease and anticipated return.

Such intermediate objects may be read, in one respect, as confirming Sadegh-Zadeh’s claim that disease concepts and disease entities often have fuzzy rather than sharply bounded extensions (Sadegh-Zadeh 1999, 2000). Yet precision medicine adds a further difficulty. The issue is not only that disease, non-disease, remission, relapse, and risk may shade into one another. It is that molecular detection, predictive modeling, and stratification for action generate clinically authoritative objects whose status may remain conceptually unstable even when they are therapeutically consequential. Ontological deskilling concerns this latter problem: the clinician must judge objects that are actionable without always being assimilable to inherited categories of disease recognition.

The philosophical significance of MRD is that it makes visible a broader feature of precision medicine. Detection does not simply reveal an object that was already clinically available. It helps determine the level at which an object becomes actionable. As assays become more sensitive, the boundary between remission and disease is not merely moved earlier; it is reconceived through thresholds, probabilities, and therapeutic possibilities. The clinician must therefore decide what kind of entity a residual signal is: evidence of present disease, a marker of future risk, a trigger for intervention, or a technologically produced intermediate state.

MRD points to a broader temporalization of disease. Disease is increasingly apprehended not only as a present pathological state to be diagnosed and treated, but as a trajectory to be anticipated, monitored, and recalculated. Wearable sensors, digital biomarkers, imaging pipelines, AI-driven progression models, and digital twins extend clinical attention toward subtle longitudinal changes, predicted transitions, and possible future states (Jensen et al. 2014; Katsoulakis et al. 2024; Morris et al. 2024; Porciuncula et al. 2018; Young et al. 2024). The relevant object becomes not only what the patient has, but where the patient is situated within a temporal sequence.

Hacking’s analysis of “making up people” and “moving targets” helps to clarify this shift without absorbing the argument (Hacking 1986, 1995, 2007). The carrier of a pathogenic variant, the person at elevated polygenic risk, or the patient with detectable MRD may inhabit a new anticipatory identity structured by surveillance, reproductive decisions, preventive intervention, or familial implication. Yet the movement at issue here is not only a looping effect in which classified persons respond to classification. It is also a biomedical-computational movement in the object itself: the level at which disease becomes clinically real is altered by molecular detection, predictive modeling, and therapeutic actionability.

## Computational representation and semantic attenuation

The transformation of the disease object does not occur in a representational vacuum. Post-genomic biomedicine produces data too heterogeneous and voluminous to be organized by traditional nomenclature alone. Genomic variants, phenotypes, biomarkers, clinical outcomes, drug responses, imaging features, and electronic health record data must be searchable, interoperable, and computable. Computational ontologies such as ICD-11, SNOMED CT, and MONDO provide part of this infrastructure. They are not merely vocabularies for naming disease; they encode biomedical entities and relations in machine-readable form (Chute and Çelik 2022; Haendel, Chute, and Robinson 2018).

The term ontology is therefore doing double work. In philosophy, it concerns what kinds of things there are and how they exist. In biomedical informatics, it refers to formal systems that represent entities and relations for computational use. Precision medicine brings these senses together. Decisions about which relations to formalize, which levels of granularity to support, and which entities to treat as equivalent or distinct are also decisions about what kinds of disease objects can circulate through research, clinical practice, reimbursement, regulation, and algorithmic reasoning (Bowker and Star 1999). What cannot be represented with sufficient precision may struggle to become an object of systematic inquiry or institutional action.

ICD-11 makes this point in institutional form by distinguishing a foundation component from different linearizations, so that a shared conceptual space can be projected differently for mortality, morbidity, primary care, or specialty use (Chute and Çelik 2022). MONDO seeks to integrate disease concepts across heterogeneous biomedical resources and thereby supports cross-database comparison and computational reasoning (Haendel, Chute, and Robinson 2018). These systems are indispensable. But they also show that classification is no longer a single hierarchy of disease names. It is a formal architecture that makes some relations computable, some distinctions actionable, and some objects institutionally mobile.

This mobility is clinically consequential. Once an object can be coded, linked, compared, and queried across infrastructures, it can acquire authority independently of the older clinical scripts through which physicians learned to recognize disease. The computational object may therefore precede the clinical object rather than merely record it. For the physician, the task is not only to interpret a disease already stabilized in clinical practice, but also to interpret objects whose stability has been conferred by infrastructures of representation, aggregation, and decision support.

Knowledge graphs and network medicine further shift disease from bounded entity to relational disturbance. Biomedical knowledge graphs integrate genes, variants, phenotypes, pathways, drugs, diseases, and outcomes into traversable networks of typed relations (Chandak et al. 2023; Himmelstein et al. 2017). Network medicine reconceives disease as perturbation within the interactome rather than as a discrete lesion located in an organ or tissue; disease modules may overlap, share molecular neighborhoods, or connect conditions previously separated by classical taxonomy (Barabási et al. 2011; Menche et al. 2015). The point is again not finer resolution alone. It is a change in the relations through which disease identity is established.

Artificial intelligence enters this landscape unevenly. Symbolic systems remain relatively close to computational ontologies because they operate through explicit concepts and relations. Deep-learning systems classify through high-dimensional statistical patterns whose relation to established biomedical categories may remain opaque. Neurosymbolic approaches attempt to bridge this divide by incorporating ontological structures into neural architectures or constraining neural outputs through symbolic knowledge (Alshahrani et al. 2017; Garcez and Lamb 2023; Kulmanov et al. 2022). Their emergence confirms rather than eliminates the tension between semantic representation and statistical pattern recognition.

This tension is clearest in algorithmic stratification. Machine-learning systems may generate clinically useful subgroups without producing stable disease classes. Patients are sorted into groups whose boundaries are predictive and actionable, but not necessarily causally interpretable, phenomenologically meaningful, or situated within a familiar differential diagnostic field. Durán and Jongsma’s concerns about trust in medical AI are relevant here: algorithmic outputs may be clinically authoritative while lacking the epistemic goods required for explanation and responsible judgment (Durán and Jongsma 2021).

The result is better described as semantic attenuation than semantic loss. Algorithmic and molecular classifications may add information, improve therapeutic targeting, and reveal previously hidden structure. At the same time, they may attenuate three forms of clinical meaning. First, causal attenuation occurs when a subgroup predicts an outcome without representing the causal structure needed for counterfactual reasoning (Pearl 2009; Holzinger et al. 2019). Second, relational attenuation occurs when a cluster lacks the contrastive position that gives a classical diagnosis meaning within a differential diagnostic field. Third, phenomenological attenuation occurs when a category fails to mediate between biological abnormality and the patient’s lived experience of illness (Carel 2016).

This is why the argument should not be read as anti-computational. The objects generated by computational systems may be clinically useful precisely because they are not constrained by inherited nosological boundaries. The problem is not their uselessness but their partiality. They may be operationally strong while remaining semantically thin; they may guide action without offering the kind of explanation, contrast, or narrative integration that clinical judgment often requires. Ontological deskilling arises when clinicians are expected to act on such objects without having been trained to interpret their specific kind of clinical reality.

Put in Canguilhemian terms, the problem is the separation of biological abnormality from lived normativity. A computationally generated subgroup may identify a statistically or therapeutically relevant pattern without corresponding to any modification in the patient’s lived capacity to inhabit a form of life. Such a category is not false. It may be clinically indispensable. But it remains normatively thin unless it can be related to the patient’s illness experience, values, temporal horizon, and practical possibilities (Abettan and Welie 2020; Canguilhem 1991; Carel 2016).

Mol’s account of ontology in medical practice helps clarify this point. In The Body Multiple, Mol argues that disease is not merely viewed from different professional perspectives; it is enacted differently across the practices through which it is examined, measured, discussed, treated, and lived. Atherosclerosis is not one object represented in many ways but a multiple object coordinated across sites of practice (Mol 2002). The present argument builds on this insight but shifts the focus. Ontological deskilling arises not simply because disease is multiple, but because the forms of multiplicity generated by molecular stratification, computational ontology, and algorithmic grouping may no longer correspond to the stabilized disease objects through which clinical perception and judgment were trained.

## An epistemically heterogeneous nosological landscape

The clinician now confronts, within the same clinical space, objects of different epistemic provenance. Some retain the semantic structure of classical nosology: causal content, relational position within a differential diagnostic field, and phenomenological resonance with illness experience. Others carry the mechanistic depth of molecular reclassification while shedding classical boundaries. Still others are algorithmically generated, operationally effective, and causally opaque. The result is not simply a more complex nosology but an epistemically heterogeneous landscape. The objects of clinical attention differ not only in content but in kind.

Molecular tumor boards provide a concrete institutional site in which this problem becomes visible. In precision oncology, clinicians, molecular pathologists, geneticists, bioinformaticians, trial experts, and others interpret genomic findings and translate them into therapeutic recommendations. Bourret and Cambrosio describe these boards as producing genomic expertise in action, where expertise is not simply possessed by an individual clinician but emerges through platforms, databases, professional roles, and interpretive practices (Bourret and Cambrosio 2019). For the present argument, their significance lies in the objects they require clinicians to coordinate: histological diagnosis, molecular alteration, variant classification, druggability, trial eligibility, regulatory access, and therapeutic actionability. These are not merely additional data attached to a diagnosis. They are heterogeneous clinical objects whose relations must be assembled case by case.

This coordination becomes strained as the epistemic distance between objects grows. An ICD code, a molecular subtype, and an algorithmic risk score are not simply different perspectives on the same disease. They are generated by different methods, governed by different validity conditions, and endowed with different forms of authority. Yet clinical practice must hold them together. When the gap between a classical diagnosis and an algorithmically generated subgroup becomes sufficiently wide, coordination itself becomes a site of epistemic labor. The clinician may be asked to act without having a stable meta-vocabulary for adjudicating among the objects before her.

This is what Cambrosio, Campbell, and Bourret call a movement “beyond nosology” in molecular tumor boards: diagnosis and therapy become partially conflated, because what the patient “has” is increasingly articulated through what is actionable, targetable, or experimentally available (Cambrosio et al. 2021). The physician is no longer only asked to recognize a disease object. She is asked to coordinate an assemblage in which diagnostic identity, therapeutic possibility, regulatory access, and evidential uncertainty are co-produced.

The resulting rupture is graded rather than total. Classical categories do not disappear. They remain necessary for communication, billing, institutional organization, epidemiology, and many forms of clinical reasoning. But they coexist with molecular, computational, predictive, and actionable objects that do not map neatly onto them. This superposition is asynchronous across clinical domains and partially buffered by institutional inertia. The clinician practices in the gap between what biomedical science reveals, what computational systems can represent, what institutions can accommodate, and what inherited clinical competence can recognize.

The term “graded rupture” avoids two misleading extremes. One extreme would claim that precision medicine has replaced classical nosology. It has not. The other would treat precision medicine as a set of refinements that leave the cognitive structure of clinical practice unchanged. That too is inadequate. The more plausible position is that medicine now contains overlapping regimes of objecthood: classical diagnoses, molecular subtypes, risk trajectories, computational clusters, and actionable alterations coexist without being reducible to a single hierarchy. The clinician’s task increasingly consists in coordinating these regimes rather than merely applying one of them.

This coordination is not a temporary inconvenience awaiting a final taxonomy. In many areas, the relevant grouping depends on the purpose of inquiry: explanation, prediction, treatment selection, surveillance, access to trials, or communication with the patient. Stability may therefore be local and provisional rather than global.

This formulation also identifies where the concept could be studied empirically. Ontological deskilling should be most visible where clinicians must coordinate objects with different provenance and authority: molecular tumor boards, genomic counseling, MRD-guided hematology, tissue-agnostic oncology, polygenic risk assessment, AI-supported prognostic modeling, and digital-twin-based monitoring. In these settings, the relevant question is not only whether clinicians know enough molecular biology or trust algorithms too much. It is whether they can translate among disease objects that differ in source, evidential status, temporal orientation, actionability, and relation to the patient’s illness experience.

## Ontological deskilling: definition and scope

Against this background, deskilling can no longer be understood only as a function of the physician-machine relationship. It must also be understood in relation to the transformation of the disease object itself. Existing accounts of technological deskilling typically ask what happens when machines perform tasks, reshape workflows, or influence judgment. Ontological deskilling asks what happens when the object toward which clinical skill is directed has changed.

Ontological deskilling occurs when competence trained in relation to inherited disease categories becomes insufficient because the clinical object has been reconstituted—molecularly, computationally, temporally, or institutionally—so that the old competence no longer fits the new object. This does not require the absence of technological deskilling. The two forms are analytically distinct but clinically cumulative: ontological deskilling may occur without technological displacement, but it may also be superimposed on technological deskilling when AI systems both mediate judgment and help stabilize newly actionable objects. The physician has not simply forgotten how to reason, nor has a machine merely displaced her skill. A skill acquired in relation to one kind of object may now be exercised in relation to another.

The word “insufficient” is important. Ontological deskilling does not imply that inherited expertise becomes worthless. On the contrary, the older competence remains necessary: clinicians still need pattern recognition, differential diagnosis, therapeutic judgment, and communicative skill. What changes is the adequacy of that competence when it is applied without second-order awareness of the object’s transformation. The problem is therefore not the disappearance of expertise but its partial decoupling from the objects that now organize clinical action.

The concept is best understood, at this stage, as a sensitizing concept rather than a psychometric construct. In Blumer’s sense, it directs attention toward a class of situations that may otherwise remain invisible (Blumer 1954; Bowen 2006). Operationally, ontological deskilling may be suspected when three conditions coincide: the clinician’s difficulty is not adequately explained by lack of practice, missing information, or erroneous algorithmic advice alone; the disease object has been molecularly, computationally, temporally, or institutionally reconstituted; and the difficulty arises primarily from a failure of fit between trained categories and the object now requiring judgment. The clinician’s older competence may remain substantially intact, but it no longer provides sufficient orientation.

It is important to specify what ontological deskilling is not. It is not simply diagnostic uncertainty, because the problem is not only that the physician lacks information about which diagnosis applies; it is that the object to which diagnostic categories refer may no longer have the categorical form presupposed by the question. It is not overdiagnosis, because the issue is not only whether an abnormality will cause harm or whether detection benefits the patient. It is not medicalization, because the central concern is not only the expansion of medicine’s jurisdiction. It is not plastic diagnostics, although it depends on diagnostic plasticity, because it concerns the competence-effects of plasticity rather than classification change itself. Nor is it simply a failure of adaptive expertise: adaptive expertise names the capacity required to respond, whereas ontological deskilling names the condition to which that capacity must respond.

This negative delimitation is necessary because the concept would otherwise become too capacious. Not every hard case, ambiguous result, novel biomarker, or AI recommendation produces ontological deskilling. The concept applies only when the clinician’s difficulty arises from the mismatch between a trained object-frame and a reconstituted clinical object. A physician who lacks the latest guideline is under-informed. A physician who cannot perform a task without software may be technologically deskilled. A physician who over-treats a harmless abnormality may be participating in overdiagnosis. Ontological deskilling names a different situation: the physician’s competence remains real, but the object to which it is directed has shifted.

These negative delimitations clarify what ontological deskilling is not in relation to adjacent clinical concerns. They leave open a further question of conceptual choice. The argument is not that traditional clinical skills become irrelevant in precision medicine. Why, then, describe the situation as a form of deskilling rather than as skill obsolescence or a shift in capabilities?

Skill obsolescence describes a capacity that is no longer required because the task to which it applied has disappeared or has been definitively replaced. The capacity may be lost without significant clinical consequence, since its conditions of relevance have ceased to obtain. Shift in capabilities describes a related but distinct situation in which one set of competencies is replaced by another better suited to a transformed task, with no residual demand for the old. In both cases the underlying assumption is that the relationship between competence and task is, in principle, restorable, whether by acknowledging that the old capacity is no longer needed, or by replacing it with the appropriate new one.

Ontological deskilling fits neither description. Inherited diagnostic competence is not superseded, since pattern recognition, differential reasoning, and therapeutic judgment remain necessary as long as classical disease categories continue to function institutionally, regulatorily, and communicatively, even when they are no longer the primary targets of intervention. Nor can this competence be cleanly replaced, because the very conditions under which expertise is acquired through recurrent engagement with relatively stable objects are what precision medicine destabilizes. The clinician therefore stands neither in a position where an old skill has become superfluous, nor in one where a definite new skill is waiting to take its place. She continues to need her trained competence while finding it insufficient for objects whose ongoing reconstitution forestalls the stabilization on which any future expertise would have to rest. This is what justifies the term deskilling. It names a structural mismatch between necessary competence and transformed object, not the redundancy of an inherited capacity nor an orderly transition from one skill set to another. The choice of term recalls its sociological origins, where deskilling described the loss of substantive control over a labor process whose labor itself continued to be required (Braverman 1974).

The distinction can be clarified through phenomenology. Clinical skill is not directed at data in general; it is directed at structured objects of recognition and action. In the phenomenological tradition, intentionality designates the directedness of cognitive acts toward objects (Brentano 1874; Husserl 1900/1901). The experienced clinician who identifies a case of type 2 diabetes, systemic lupus erythematosus, or breast cancer is not merely processing variables. She is engaging an object that carries a history, a differential field, a prognostic horizon, and a practical orientation. If that object is reconstituted, the cognitive act may retain its form while losing reliable correspondence with its target.

Technological and ontological deskilling are therefore distinguishable but not mutually exclusive. In technological deskilling, the intentional object may remain relatively stable while the cognitive task is delegated, distorted, or insufficiently exercised. In ontological deskilling, the object itself changes: the disease category on which the physician was trained is decomposed, realigned, or supplemented by objects of different epistemic kind. In contemporary practice these processes may overlap. A clinician may depend on an AI system not only because a task has been automated, but because the system is helping to constitute or authorize an object that inherited clinical categories do not make fully intelligible. The physician’s cognitive act retains its direction, but the clinical world no longer answers it in the same way.

This is why ontological deskilling is not merely the familiar problem of keeping up with new knowledge. Continuing medical education can transmit new facts, guidelines, and classifications. What it cannot easily transmit is a new mode of perceptual engagement with a domain whose objects no longer present themselves as stable, bounded, and categorically distinct. The issue is not that the physician knows too little. It is that the physician may know in a way that no longer fits the object to which that knowledge is applied.

Dreyfus’s account of expertise makes the point more precise. Expert performance depends on the gradual formation of immediate, situation-sensitive recognition through repeated engagement with a domain (Dreyfus and Dreyfus 1986). Such recognition presupposes a world that recurs in sufficiently stable patterns. When the objects of a domain are reorganized, the expert’s perceptual fluency may become a liability. A clinician who has developed an immediate grasp of systemic lupus erythematosus as a clinical entity may face difficulty when the category is molecularly decomposed into partially overlapping immunological processes with divergent prognostic and therapeutic implications (Banchereau et al. 2016). The problem is not the absence of expertise, but its possible misalignment with a transformed object.

This point should be distinguished from the banal claim that expertise must be updated. Experts can and do learn new mechanisms, guidelines, and taxonomic revisions. The deeper difficulty is that pattern recognition is trained through recurrence. If the object becomes context-dependent, purpose-relative, and recurrently restratified, the conditions under which expert perception develops are themselves altered. The clinician is not simply asked to add a new pattern to an existing repertoire. She is asked to recognize when the repertoire’s underlying object-structure is no longer stable enough to support the same mode of recognition.

Heidegger’s distinction between the ready-to-hand and the present-at-hand illuminates the experience of this misalignment, provided it is used modestly. In ordinary clinical work, nosological categories function transparently: the physician thinks through them rather than about them. When those categories fracture, they lose this transparency. They become objects of reflection, hesitation, and repair. The category that previously organized the field now appears as an instrument whose reliability can no longer be taken for granted (Heidegger 1927).

Four markers can help distinguish ontological deskilling from ordinary diagnostic difficulty. First, performance remains competent when patients are understood within inherited diagnostic categories. Second, disorientation appears when patients previously treated as instances of the same disease category are separated by molecular subtype, actionable variant, MRD status, risk trajectory, or algorithmic subgroup. Third, the clinician struggles to translate among an ICD diagnosis, biomarker profile, risk score, and therapeutic actionability. Fourth, subjective confidence persists even when the illness script no longer matches the epistemic form of the object now requiring judgment. These markers do not prove the existence of a new psychological deficit. They identify a disruption in the relation between clinical competence and its object.

Consider how this might appear in practice. A clinician may be fully competent in diagnosing and staging a malignancy according to conventional categories, yet uncertain how to interpret a molecular alteration that makes the patient eligible for a tissue-agnostic therapy, a trial, or an off-label intervention. Another may understand remission in the classical sense but struggle to explain the clinical status of MRD positivity: not relapse, not cure, not simple risk, but a residual signal whose significance depends on assay sensitivity, disease context, and therapeutic options. In such cases the difficulty is not merely that the clinician lacks a fact. It is that the clinical object no longer has the form around which the relevant competence was organized.

## Implications for clinical judgment and responsibility

Ontological deskilling has practical consequences because clinical judgment depends on more than the accumulation of facts. Classical expertise within categorical medicine rested on a loose but powerful alignment among three expectations: that there is a determinate object from which the patient suffers, that this object can in principle be recognized, and that recognition can guide action. These expectations were never absolute. Medicine has always involved uncertainty. But they gave routine clinical reasoning its working architecture under conditions of incomplete information and time pressure (Schmidt and Rikers 2007; Schwartzstein 2024).

These expectations should be understood as regulative conditions of practice rather than philosophical certainties. They allow physicians to proceed as if the disease object, the act of recognition, and the therapeutic pathway are sufficiently aligned for responsible action. Much of the efficiency of clinical reasoning depends on this practical compression. The physician does not ordinarily ask what kind of object diabetes, breast cancer, or lupus is before acting. She works through categories whose stability allows the question of ontology to remain tacit.

Precision medicine weakens all three expectations. The certainty of object is weakened when a disease category is revealed as a collection of molecularly distinct processes. The certainty of recognition is weakened when a patient’s position is distributed across molecular, phenotypic, probabilistic, and algorithmic spaces rather than located within a single category. The certainty of action is weakened when the same molecular profile can support multiple therapeutic options, none of which carries the determinacy associated with classical diagnosis-treatment scripts (Alyass et al. 2015; Hager et al. 2024).

The earlier discussion of semantic attenuation maps onto this structure. Causal attenuation weakens object certainty because a predictive grouping may not disclose the causal organization of the disease. Relational attenuation weakens recognition because an algorithmic cluster may not occupy a stable position within a differential diagnostic field. Phenomenological attenuation weakens action because clinical action includes explanation, communication, and the construction of a shared narrative with the patient, not only the selection of an intervention (Carel 2016).

This transformation modifies the nature of uncertainty. The uncertainty generated by precision medicine has already been described in terms of evidence, implementation, communication, ethics, and translation (Lohse 2023). Vogt’s “precision paradox” captures the way more individualized and data-rich medicine may produce rather than reduce uncertainty (Vogt 2022). Borck and Lohse further distinguish socio-technical, epistemological, and ontological sources of uncertainty in precision medicine (Borck and Lohse 2026). The present argument specifies a narrower mechanism: uncertainty becomes clinically destabilizing when the disease object no longer has the stable form presupposed by the physician’s trained categories of recognition and action.

Within the categorical framework, uncertainty was primarily epistemological: the physician did not yet know which category applied, but further inquiry might close the gap. In ontological deskilling, uncertainty can be object-level. The problem is not only that the answer has not yet been found; it is that the question may presuppose a stable object that the clinical situation no longer contains. Further investigation may reveal additional heterogeneity rather than convergence. The difficulty is not simply that the answer is elusive, but that the diagnostic question may no longer match the structure of what it interrogates (Han and Hofmann 2026; Trachana et al. 2018).

This does not mean that all uncertainty in precision medicine is ontological. Some uncertainty remains ordinary and informational: the test is missing, the evidence is weak, the image is equivocal, or the history is incomplete. Other uncertainty is taxonomic: the available categories do not organize the phenomena well. Still other uncertainty is intrinsic: biological complexity may resist the stable partitioning that classification seeks. Ontological deskilling becomes clinically salient when physicians interpret all three as if they were merely informational and therefore seek resolution only through more data, more tests, or more algorithmic support.

This condition also produces an illusion of expertise. The term does not imply pretension or negligence. It names a structural mismatch between the subjective experience of competence and the actual fit between competence and object. In technological deskilling, the loss of skill may eventually be detected: the physician has ceased to perform a task and performs worse when assistance is removed. In ontological deskilling, the skill may remain subjectively intact. The clinician continues to recognize patterns, formulate differentials, and act with confidence, while the objects those patterns once tracked have been reconfigured.

The danger is therefore greatest where expertise feels most secure. A novice may know that she is uncertain; an expert may experience fluency precisely because her cognitive architecture has been deeply shaped by inherited categories. When those categories are displaced, fluency can conceal inadequacy. This is not an indictment of experts. It is a structural feature of expertise under conditions of object transformation.

The automation-bias literature provides indirect evidence of how such misalignment may appear. The Goh et al. trial suggests that strong AI performance does not automatically translate into improved physician performance; the problem is one of integration as much as information (Goh et al. 2024). When algorithmic outputs arise from epistemic structures unlike those in which clinicians were trained, physicians may lack the categories needed to evaluate their meaning. The illusion of explanatory depth thereby acquires a clinical form: clinicians may overestimate not only their grasp of a mechanism, but the adequacy of their cognitive framework to a transformed disease landscape (Rozenblit and Keil 2002).

The ethical implications follow from this epistemic structure. If the uncertainty confronting the physician arises partly from the transformation of the disease object, responsibility cannot be grounded only in the expectation of correct recognition. The physician is not absolved of responsibility, but its ground shifts. Responsibility becomes less a matter of simply “getting it right” within an established taxonomy and more a matter of judging well when the stability of the taxonomy itself is at issue (Cutter 2024; Pellegrino and Thomasma 1993).

The normative weight of this shift exceeds responsibility for individual decisions. Three implications follow. First, the trustworthiness of the clinical encounter depends on the physician’s capacity to recognize the limits of her own framework. The patient’s reasonable expectation is not that the physician knows everything, but that she can identify when the conditions of her competence apply and when they may not. Ontological deskilling threatens this second-order capacity precisely by producing fluency without fit. Second, informed consent in the face of reconstituted disease objects depends on the physician’s ability to convey what kind of object is being acted upon. When an algorithmic subgroup, a residual signal, or a risk state is communicated as if it were a classical diagnosis, the patient is asked to consent to interventions whose object-status has not been made transparent. Phenomenological attenuation is therefore not only an epistemic shortcoming. It is a normative shortcoming, because the patient’s capacity to deliberate and decide depends on receiving an object she can recognize as bearing on her life. Third, the burdens of object transformation cannot fairly be placed on individual clinicians alone. A clinician misaligned with a reconstituted disease object is not simply underprepared, but operates in a clinical world whose objects have been altered by scientific, computational, and institutional developments over which she has had little control. To frame the problem as a matter of personal updating would be to make individuals bear the moral cost of a structural condition.

This is why the appropriate response cannot consist only in acquiring new skills and moving forward. Additional information, further certifications, and familiarity with new tools are necessary but insufficient. The situation calls for a transformation of the conditions under which clinical competence is acquired, exercised, and evaluated. These conditions include medical curricula that thematize the contingency of nosological categories, institutional settings in which the multiplicity of disease objects becomes a routine topic of clinical conversation, regulatory frameworks attentive to the epistemic novelty of algorithmically constituted objects, and forms of professional development that treat reflexivity about the disease object as part of expertise rather than an addition to it. The normative significance of ontological deskilling is therefore double. It identifies a structural condition that exceeds individual responsibility, and it places obligations on institutions, regulators, and educators that cannot be discharged simply by asking clinicians to keep learning.

Ontological deskilling also differs from overdiagnosis at the ethical level. The ethics of overdiagnosis focuses on avoidable harm: unnecessary labeling, treatment, anxiety, surveillance, and cost. Ontological deskilling adds another responsibility: the physician must recognize when the classificatory object through which she explains, predicts, and acts no longer has the form presupposed by inherited diagnostic competence. The ethical demand is therefore not only restraint in the face of medical expansion, but reflexivity in the face of transformed disease objects.

Communication with patients is directly affected. To tell a person that she is a carrier, at elevated algorithmic risk, MRD-positive, or a member of an actionable subgroup is not merely to report a result. It may place her within an anticipatory identity organized around surveillance, preventive obligation, familial implication, or uncertain future disease (Hacking 1986, 2007). The physician must translate an operational disease object into terms that can be situated within the patient’s life. This translation is ethical because the significance of a risk state or molecular finding depends partly on values, plans, family relations, tolerance of uncertainty, and sense of self.

The convergence of technological and ontological deskilling intensifies the problem. The two forms need not occur together, but when they do, each amplifies the other. Technological dependence becomes more dangerous when the objects of clinical attention are unstable, because the physician has fewer independent resources with which to evaluate algorithmic outputs. Ontological disorientation becomes harder to recognize when algorithmic systems supply confident and operationally effective recommendations. The distinctive challenge of contemporary practice lies in this interaction: clinicians must exercise responsibility toward objects that are being reconstituted while relying on tools whose epistemic processes may be only partially inspectable.

This convergence also explains why the article begins with AI but does not treat AI as the sole cause. AI can deskill by substituting for practice or by biasing judgment, but it can also stabilize and legitimize newly constituted objects. An algorithmic subgroup, risk score, or predicted trajectory may appear clinically authoritative because it is embedded in a decision-support workflow, even when its relation to established disease categories remains unclear. The machine does not merely mediate access to a pre-existing object; in some settings it participates in making the object clinically salient. The physician must therefore evaluate both the tool and the object that the tool helps disclose.

## Modes of response

If ontological deskilling is not reducible to technological deskilling, then the usual remedies for the latter are insufficient. More unassisted practice, simulation without AI, and alternation between assisted and unassisted workflows may help preserve capacities threatened by automation (Natali et al. 2025; Oettl et al. 2026). But they presuppose that there is a stable competence to which the physician can return. A clinician who resumes unassisted reasoning within a domain whose objects have been molecularly fragmented may recover a skill whose referent has changed. Restoration is a remedy for loss; ontological deskilling requires a response to displacement.

Two features distinguish ontological deskilling at the level of response. First, the phenomenon is harder to measure than technological deskilling. Deskilling associated with clinical decision support systems in radiology or pathology can in principle be detected by comparing assisted and unassisted performance on tasks with stable referents. Ontological deskilling resists this design because the referent itself is the variable, and there is no fixed benchmark against which to measure unassisted competence, since the disease object on which that competence was trained is what has been displaced. Second, the phenomenon operates at a more foundational level than performance on discrete tasks. It concerns the conditions under which clinical perception, reasoning, and communication are organized, rather than the success or failure of any particular act. The implications for response are immediate. Remedies that address technological deskilling typically take the form of training intensification or workflow design. Remedies adequate to ontological deskilling must intervene on the conditions of expertise itself, including how categories are taught, how multiplicity of objects is institutionalized in clinical practice, and how the tools that participate in object-construction are designed and regulated.

The obvious educational response is adaptive expertise. Adaptive expertise is usually contrasted with routine expertise: routine experts perform efficiently in familiar situations, whereas adaptive experts modify and reorganize knowledge when confronted with novelty, complexity, or changing conditions (Hatano and Inagaki 1986; Mylopoulos and Woods 2017). Ontological deskilling sharpens this claim. The relevant novelty is not simply an unfamiliar patient or context, but a transformation of the disease object itself.

This also prevents a possible misunderstanding of the argument. Ontological deskilling does not mean that physicians should abandon routine expertise or cultivate permanent skepticism about every diagnosis. Routine expertise is indispensable in most clinical settings. The problem is not routine expertise as such, but routine expertise without the capacity to detect when the object has ceased to be routine. Adaptive expertise supplies the educational vocabulary for that capacity; critical nosological awareness specifies its domain-specific content.

The needed response is therefore not an increase in the quantity of clinical knowledge but a change in its order. First-order capacity remains indispensable: physicians must recognize, diagnose, and act within disease categories. Second-order capacity is epistemological: physicians must understand that the categories through which they reason are historically constituted, institutionally embedded, and ontologically mutable. Third-order capacity is critical and reflexive: physicians must recognize that categories serve multiple functions—clinical, research, regulatory, financial, communicative, and social—that may not always align.

Critical nosological awareness designates the conjunction of second- and third-order capacities. It is the ability to reason clinically within disease categories while also recognizing their contingency, multiplicity of function, and susceptibility to transformation. It does not replace routine clinical competence. It presupposes it. But it changes the physician’s relation to that competence by making visible what routine expertise tends to conceal: the conditions under which categories were stabilized and the circumstances under which they may fail.

This awareness is not a call to turn clinicians into professional philosophers. It is a clinical virtue with philosophical content. It asks physicians to know when a category is functioning as a diagnostic label, a research grouping, a reimbursement code, a therapeutic proxy, or a patient identity. These functions may overlap, but they need not coincide. The physician who cannot distinguish them risks treating an institutional or algorithmic object as if it were a clinically transparent disease entity.

In this sense, critical nosological awareness is a domain-specific form of adaptive expertise. Its aim is not to replace efficient recognition with endless reflection, but to cultivate the capacity to move between fluency and critique when the object no longer fits inherited scripts (Mylopoulos et al. 2018; Pusic et al. 2018). A clinician with only first-order competence may experience ontological displacement as confusion, diagnostic difficulty, or personal inadequacy. A clinician with critical nosological awareness can identify the source of difficulty as a transformation in the object of judgment.

This capacity cannot be acquired by bypassing ordinary nosological training. The physician who has not internalized disease categories cannot meaningfully reflect on their contingency. The pedagogical problem is therefore one of sequencing and integration. Medical education must cultivate enough routine expertise for safe, rapid recognition, while preventing that routine expertise from becoming dependent on the false expectation that disease categories are fixed.

This is the central pedagogical paradox. Categories must be taught as reliable enough to support action, but revisable enough to remain open to transformation. If they are taught only as stable entities, students acquire fluency at the cost of rigidity. If they are taught only as historically contingent constructs, students may lose the practical confidence needed for clinical work. Education adequate to ontological deskilling must therefore teach categories as instruments: indispensable, powerful, and historically formed, but not identical with the clinical world itself.

One practical strategy is to teach nosological scripts together with revision scripts. Students should learn not only what a disease category currently does, but how it has changed, what it presupposes, what it excludes, and what follows when those presuppositions fail. The history of nosology is not a cultural ornament. It is a way of making the contingency of categories visible without undermining their practical use. A student who learns cancer staging alongside the reasons for its molecular revision acquires not only current knowledge but preparation for future learning (Mylopoulos et al. 2016; Wadmann 2023).

Curricula should therefore include variation, contrast, and productive difficulty rather than only canonical exemplars. Applied to nosology, this means teaching disease categories alongside borderline cases, failed classifications, molecular reclassifications, risk states, and competing purposes of classification. Students should be asked to compare how patients previously grouped under the same diagnosis may be redistributed as molecular stratification problems, risk-management problems, therapeutic-actionability problems, or algorithmic prediction problems. The aim is not to weaken diagnostic confidence, but to distinguish confidence that is warranted by stable objects from confidence that merely reflects familiarity with inherited scripts.

This requirement extends to epistemic literacy in relation to artificial intelligence. Physicians need to understand not only what an AI system does, but what kind of object it constructs, what ontology or data structure it presupposes, and where its outputs may be semantically thin. Such literacy is not merely technical. It concerns the ability to ask whether an algorithmic output is a diagnosis, a risk estimate, a cluster, a treatment proxy, or an institutional classification. Without this distinction, the physician may believe she is deliberating when she is largely conforming to an object whose epistemic status she cannot inspect (Babic et al. 2021).

The institutional dimension is equally important. If assessment systems reward only convergence on a single answer, they will reinforce the very cognitive disposition that ontological deskilling makes fragile. Multiple-choice examinations, competency assessments, tumor board documentation, and clinical governance structures should evaluate not only accuracy but also the quality of reasoning across heterogeneous objects. Clinicians should be rewarded for distinguishing informational uncertainty from taxonomic or ontological uncertainty, for explaining the evidential status of molecular or algorithmic findings, and for communicating uncertainty without collapsing it into ignorance (Han and Hofmann 2026; Simpkin and Schwartzstein 2016; Stephens et al. 2021).

Concrete settings for such training already exist. Molecular tumor boards can be used not only to generate recommendations but to teach how histology, molecular alteration, trial eligibility, drug access, and patient goals are coordinated. Genomic counseling can make explicit the difference between present disease, probabilistic risk, familial implication, and anticipatory identity. MRD-guided hematology can teach the status of residual signal as neither ordinary disease nor simple remission. AI-supported prognostic settings can require clinicians to state whether an output is causal, predictive, classificatory, or merely operational. These are not additions to clinical reasoning; they are exercises in reasoning about the changing objects of clinical care.

Advanced training and continuing professional development require their own attention, distinct from the design of initial medical curricula. Mid-career and senior clinicians are not in the position of trainees acquiring expertise for the first time. Their cognitive architecture has been shaped, often deeply, by the nosological regime in which they were formed. The illusion of expertise discussed earlier is most likely to affect precisely this group, because their fluency was acquired within categories that may no longer organize the object. Continuing professional development should therefore not be conceived simply as the transmission of new content. It should include structured opportunities to revisit the conceptual scaffolding of one’s own training, asking which categories were treated as stable, how those categories are now being reorganized, and what the implications are for one’s working clinical ontology. This is not retraining in the ordinary sense. It is a form of reflective practice that takes the contingency of one’s earlier formation as an object of professional development in its own right.

The reform required is thus structural as well as curricular. It concerns what is taught, how competence is assessed, and what forms of reasoning institutions reward. An education adequate to ontological deskilling would cultivate epistemic resilience: the capacity to maintain sound clinical judgment when the objects of judgment are themselves undergoing transformation. This is not a call to abandon diagnostic precision. It is a call for a more reflective relationship to the conditions under which precision is achievable and to the responsibilities that arise when it is not.

Several modest institutional changes would follow. Case conferences could require explicit statements of the object under discussion: classical diagnosis, molecular mechanism, risk state, therapeutic target, or algorithmic prediction. Assessment tasks could ask students to explain how patients previously treated as belonging to the same disease category may become clinically different when stratified at molecular, temporal, or algorithmic levels. Clinical documentation could distinguish evidential uncertainty from taxonomic uncertainty rather than recording all doubt as insufficient information. Decision-support interfaces could make visible whether a recommendation is grounded in causal evidence, statistical association, guideline consensus, or trial eligibility. None of these measures eliminates ontological deskilling, but each makes the underlying object-relation more inspectable.

AI development itself is implicated in the response. If algorithmic systems participate in the constitution of new clinical objects, then their design is not normatively neutral with respect to ontological deskilling. Several design principles follow. AI systems used in clinical practice should make explicit the kind of object their outputs constitute, distinguishing for example between a probabilistic risk estimate, a predictive cluster, a diagnostic suggestion calibrated against established categories, and an algorithmic grouping with no direct referent in classical nosology. They should expose the data structures and ontological commitments through which their outputs are produced, so that clinicians can evaluate not only the recommendation but its object-status. Where this is impossible in practice, the limitation should be visible to the user rather than concealed by interface design. Regulatory frameworks have a complementary role. Standards for clinical AI should require, in addition to performance metrics, transparency about the kind of object an algorithm constructs and the relation between that object and the categories within which clinicians have been trained. None of this eliminates ontological deskilling. It does, however, prevent algorithmic systems from compounding the phenomenon by presenting computationally constituted objects as if they were diagnoses of the classical kind.

## Conclusion

This article has argued that contemporary discussions of physician deskilling, while valuable, are limited when they focus only on the effects of intelligent systems on clinical capacities. Precision medicine changes not only the tools of clinical reasoning but also the disease objects toward which reasoning is directed. Established categories are fragmented, realigned, temporalized, computationally represented, and supplemented by actionable risk states and algorithmic subgroups. Under these conditions, clinical competence may remain intact and nevertheless become insufficient.

Ontological deskilling names this failure of fit between inherited competence and transformed object. Technological deskilling is primarily a problem of substitution, dependence, or distorted performance. Ontological deskilling is a problem of reference: the physician’s cognitive act persists, but the object toward which it is directed has been reconstituted. The two problems are analytically distinct, not empirically exclusive; in contemporary precision medicine they may be layered upon one another. The concept is not meant to replace accounts of uncertainty, medicalization, overdiagnosis, diagnostic plasticity, or adaptive expertise. It identifies the point at which these concerns converge at the level of clinical competence.

The implications extend to clinical judgment, uncertainty, and responsibility. The inherited alignment among object, recognition, and action is weakened. Uncertainty becomes not only informational but sometimes taxonomic or ontological. The illusion of expertise becomes more difficult to detect because the clinician’s skills may remain subjectively secure while their fit to the disease landscape is disrupted. Responsibility therefore requires not only correct recognition within existing categories, but reflexive judgment about the categories themselves. These implications carry normative weight beyond individual decision-making. They concern the trustworthiness of the clinical encounter, the conditions under which informed consent can be meaningfully offered, and the fair distribution of the burdens of object transformation between individual clinicians and the institutions in which they work.

The appropriate response is critical nosological awareness. Physicians must continue to inhabit disease categories with practical competence, but they must also learn to recognize their contingency, historical formation, institutional functions, and susceptibility to transformation. This capacity cannot rest on individual reflection alone. It must be supported by curricular reform that integrates the contingency of nosology into clinical training, by forms of continuing professional development that allow established clinicians to revisit the categories within which their expertise was formed, and by the design and regulation of AI systems that make explicit the kind of object their outputs constitute. None of this is philosophical ornamentation added to clinical training. It is a condition of expertise in a medicine whose objects are increasingly molecular, computational, temporal, and heterogeneous.

The argument developed here is conceptual rather than empirical, and its clinical manifestations require further study. Molecular tumor boards, genomic counseling, MRD-guided hematology, tissue-agnostic oncology, polygenic risk assessment, AI-supported prognostic modeling, and digital-twin-based monitoring are promising sites for such work. Future research could examine how clinicians translate among classical diagnoses, molecular alterations, risk states, algorithmic outputs, and therapeutic actionability. If ontological deskilling is to become empirically useful, it should be operationalized not as a measure of general clinical competence, but as a measure of judgment under conditions in which the object of judgment is unstable.

The argument is also domain-sensitive. Ontological deskilling will not appear with equal force across all areas of medicine. It is likely to be most acute where disease categories are being actively reconstituted by molecular stratification, predictive modeling, or algorithmic grouping, and less acute where categories remain clinically stable enough to sustain ordinary expertise. This qualification is important. The claim is not that all medicine is becoming ontologically unstable in the same way, but that some strategically important domains are already organized by disease objects for which inherited clinical competence is only partly adequate.

The central question is therefore not only whether physicians can maintain their skills in the presence of intelligent machines. It is whether they can maintain clinical judgment when the disease objects toward which those skills were trained are themselves in motion.

This reorientation is the article’s main contribution. It does not deny the importance of AI-induced technological deskilling, nor does it claim that precision medicine makes clinical expertise obsolete. It argues that a theory of deskilling adequate to contemporary medicine must include the object-side of expertise. Skills are not free-floating capacities. They are capacities directed toward historically formed objects. When those objects move, the preservation of skill is no longer sufficient; the relation between skill and object must itself become a matter of clinical reflection.
